# Blood markers (lymphocyte percentages, neutrophils, CRP and ESR) can help in prioritizing rRT-PCR test for suspected COVID-19 patients in countries with limited health resources

**DOI:** 10.11604/pamj.2020.37.331.25180

**Published:** 2020-12-10

**Authors:** Abd-Alhafeez Osman Ibnouf, Mohamed Hilal Khalil, Rayan Khalid, Elshibli Mohamed Elshibli, Osman Elsayed, Imad Fadl-Elmula

**Affiliations:** 1 Department of Medical Laboratory Sciences, Port Sudan Ahlia College, Port Sudan, Sudan,; 2 Department of Medical Laboratory Sciences, Omdurman Islamic University, Omdurman, Sudan,; 3 Department of Clinical Genetics, Alneelain Stem Cells Centre, Alneelain University, Khartoum, Sudan,; 4 Department of Biostatistics, Assafa Academy, Khartoum, Sudan,; 5 Department of Medicine, Faculty of Medicine, Alneelain University, Khartoum, Sudan

**Keywords:** COVID-19, blood markers, coronavirus

## Abstract

**Introduction:**

the outbreak of coronavirus disease 2019 (COVID-19) started in China in December 2019 and spread causing more than 14 million cases all over the world on July 19^th^, 2020. Although, real-time reverse transcription polymerase chain reaction (rRT-PCR) test is the gold standard test, it needs a long time and requires specialized laboratories and highly trained personnel. All these difficulties forced many countries with reduced health resources to limit rRT-PCR tests to individuals with severe symptoms. Thus, routine blood marker that may help physicians to suspect COVID-19 and hence, prioritize patients for molecular diagnosis is badly needed.

**Methods:**

fifty-six Sudanese COVID-19 patients admitted to Jabra hospital were included in this study. For all the patients we analyzed complete blood count (CBC), CBC, plasma levels of C-reactive protein (CRP), erythrocyte sedimentation rate (ESR), liver function tests (LFT) and renal function tests (RFT). Statistical analysis was done using SPSS program with a significance level of p≤0.05 and confidence limits (CLs) 95%. The difference between groups was tested using Mann-Whitney test was for quantitative variables while qualitative variables was tested using chi-square (Fisher exact) test.

**Results:**

the result shows that, 35 out of the 56 patients (62.5%) were male and 21 (37.5%) were females with a median age of 60-year-old for both sexes. Lymphocytes % showed decrease to 9.2 (P-value=0.000) and significant increase in neutrophils to 83.05 (P-value=0.005), ESR to 65.54 (P-value=0.000) and CRP to 91.07 (P-value=0.000). The receiver operating characteristic curve (ROC)/area under the curve (AUC) ensured the expellant result of lymphocytes % as a predictor with 92% area under the curve, neutrophils were 90% and ESR 95.8%. The percent of detecting COVID-19 positive RT-PCR (98%) for suspected individuals using ROC showed best cutoff of ≤21.8 for lymphocytes %, ≥67.7 for neutrophils, ≥37.5 for ESR, ≥6.2 for CRP and ≥7.15 for WBCs.

**Conclusion:**

the results also showed that, lymphocyte percentages, neutrophils, CRP and ESR may be used as markers for COVID-19 helping prioritizing individuals for rRT-PCR test.

## Introduction

A cluster of unexplained pneumonia cases were reported by the People´s Republic of China to the World Health Organization (WHO) on December 31^st^, 2019. By January 12^th^, 2020, China shared with the world the sequence of a novel virus (COVID-19) and later on the 13^th^ of January 2020, Thailand confirms the first case of COVID-19 outside of China.

The etiology for this outbreak was a new coronavirus named severe acute respiratory syndrome coronavirus 2 (SARS-CoV-2) which was responsible for the coronavirus disease 2019 (COVID-19) [1]. On July 23^rd^, 2020, the disease has spread to 213 countries worldwide with almost 15 million infected people and more than 618,017 deaths were reported to the WHO [2]. By March 13^th^, 2020, Sudan had confirmed the first case of COVID-19 infection using real time reverse transcriptase polymerase chain reaction (rRT-PCR), performed on respiratory samples of a patient that had returned from the UAE. By 24^th^ July, a total of 11,237 patients were admitted and/or confirmed as COVID-19.

The rRT-PCR test remains the gold standard method for the etiological diagnosis of COVID-19 infection. Unfortunately, the maximum benefit use of rRT-PCR methods for diagnosis of COVID-19 was hindered in many countries, especially in the developing ones, by the limitation of molecular laboratories and well-trained personnel [3]. All these difficulties forced many countries with reduced health resources to limit the available rRT-PCR tests to individuals with pronounced respiratory syndrome symptoms [4]. This policy is reflected in under diagnoses of the disease due to reduction and/or delay in the number of tested patients for COVID-19 using RT-PCR, especially in those with non-classical COVID-19 presentation, which in turn led to increase community spread of the disease. This situation is seen in most African countries e.g. Sudan has only one testing center for COVID-19 with maximum capacity of 500 RT-PCR for COVID-19 test/day in Khartoum, which has population of 6 million inhabitants. Recent studies showed that some routine blood tests markers may help in prioritizing rRT-PCR for COVID-19 suspected patients in countries with limited health resources [5]. Thus, the aim of the present study is to identify the profile of routine blood test (markers) for COVID-19 patients to be used for prioritizing RT-PCR for COVID-19 suspected patients in countries with limited resource settings.

## Methods

A total of 56 Sudanese COVID-19 patients (35 (62.5%) males and 21 (37.5%) females) admitted to Jabra Hospital, Khartoum, Sudan were included in this study. All the patients had respiratory symptoms and were tested positive for COVID-19 using rRT-PCR before admission to the isolation ward. For all patients, the complete blood count (CBC), C-reactive protein (CRP), erythrocyte sedimentation rate (ESR), total protein, albumin, total and direct bilirubin, aspartate aminotransferase (AST), alanine aminotransferase (ALT), alkaline phosphatase (ALP), urea, creatinine and electrolytes were measured on admission of the patients.

Statistical analyses were done using statistical package for social sciences (SPSS) version 21 with a significance level of p≤0.05 and CLs 95%. The descriptive statistics (mean ±SD, median) was calculated to describe quantitative variables. The median was the best central measure because of the data abnormality, so the sign test was used instead of the parametric test and qualitative variables were described using frequency and percent. The differences between frequencies were tested by goodness of the fit test using chi-square or Fisher exact test when needed. The relationships between quantitative variables were tested by spearman correlation test, receiver operating characteristic curve and area under the curve (ROC/AUC) were used to obtain the true positive and false positive predictive values calculated using the best cutoff values.

The ethical clearance for conducting this study was obtained from the Ethical Committee Board of Assafa Academy. Patients were not contacted directly; data and laboratory results were obtained from hospital archive and kept anonymous at all stages of the study.

## Results

Of the 56 patients, 35/62.5% were male and 21/37.5% were females, all were a Sudanese mixture of Nilo-Saharan and Afro-Asiatic ethnic origin and all were COVID-19 positive using rRT-PCR at the time of admission. Their age ranging between 10 - 82-year-old and the median age was 60-year-old. The males were significantly older (median 62) than the females (median 50) using Mann-Whitney test with P-value=0.003.

The results showed that the plasma median levels of lymphocytes, neutrophils, CRP, ESR, urea, Na^+^ and K^+^ were altered being higher or lower than normal (Table 1). For the red blood cells (RBCs), white blood cells (WBCs), platelets (PLTs), Hemoglobin (Hb), total protein, albumin, liver enzymes and creatinine the median values were within normal level (Table 1).

**Table 1:** descriptive statistics of blood markers among Sudanese COVID-19 patients

	Mean	Std. deviation	Median	Normal range	Comment	P-value
Age	55.09	16.50	60.00			
WBCs	10.75	5.02	9.40	4-10	N	
RBCs	4.21	0.93	4.44	M=4.5-6.5, f=3.5-5.5	N	
HGB	11.46	2.57	11.75	11-17	N	
PLT	296.57	149.96	282.50	150-450	N	
Lymphocytes %	14.45	12.48	9.20	20%-45%	L	0.000
Neutrophils %	77.03	17.51	83.05	45%-75%	H	0.005
MXD %	7.01	3.60	5.90	3%-15%	N	
Urea	51.91	54.90	28.00	8-20 mg/dL	H	0.000
Creatinine	3.93	9.79	1.00	0.6-1.2 mg/dl	N	
Total bilirubin	0.53	0.24	0.53	<1.5 mg/dL	N	
Direct bilirubin	0.28	0.20	0.27	<0.4 mg/dL	N	
Total protein	14.32	20.20	7.00	6.0-8.0 g/dL	N	
Albumin	4.42	5.25	3.20	3.5-5.5 g/dL	N	
Na	131.44	6.75	131.72	135-145 mEq/L	N	
K	6.01	9.04	3.80	3.5-5.1 mEq/L	N	
AST	60.12	62.75	36.00	7-40 mU/mL	N	
ALT	43.77	44.69	35.00	5-35 mU/mL	N	
ALP	134.28	115.51	100.50	35-100 U/L	H	
ESR	65.54	24.67	65.54	0-15 mm/h	H	0.000
CRP	91.17	72.23	91.17	<3.0 mg/L	H	0.000

The analysis showed significant frequency distribution amongst patients groups with normal, high or low values using chi-square test for lymphocytes (P=0.000), neutrophils (P=0.000), PLT (P=0.000), CRP (P=0.000), ESR (P=0.000), total protein (0.001), albumin (P=0.000), direct bilirubin (P=0.000), urea (P=0.003), creatinine (P=0.001), Na^+^ (P=0.000), and K (P=0.001) (Table 2). For the remaining variables (RBCs, Hb, WBCs, total bilirubin, AST, ALT, ALP) no statistical significance was seen amongst patients’ groups of normal, high or low values (Table 2).

**Table 2:** the frequency distribution of blood markers among Sudanese COVID-19 patients

		Count	Column N%	P-value
WBCs status	Low WBCs	0	0.0%	0.593
	Normal WBCs	30	53.6%	
	High WBCs	26	46.4%	
RBCs status	Low RBCs	25	44.6%	0.423
	Normal RBCs	31	55.4%	
	High RBCs	0	0.0%	
HGB status	Low HGB	20	35.7%	0.033
	Normal HGB	36	64.3%	
	High HGB	0	0.0%	
PLT status	Low PLTs	7	12.5%	0.000
	Normal PLTs	41	73.2%	
	High PLTs	8	14.3%	
Lymphocytes status	Low lymphocytes	44	78.6%	0.000
	Normal lymphocytes	11	19.6%	
	High lymphocytes	1	1.8%	
Neutrophils status	Low neutrophils	3	5.4%	0.000
	Normal neutrophils	14	25.0%	
	High neutrophils	39	69.6%	
MXD status	Low MIX%	8	14.3%	0.000
	Normal MIX%	47	83.9%	
	High MIX%	1	1.8%	
Urea status	Low urea	0	0.0%	0.003
	Normal urea	17	30.4%	
	High urea	39	69.6%	
Creatinine status	Low creatinine	7	12.5%	0.001
	Normal creatinine	29	51.8%	
	High creatinine	20	35.7%	
Total bilirubin status	Normal total bilirubin	56	100.0%	No
	High total bilirubin	0	0.0%	
Direct bilirubin status	Normal direct bilirubin	49	87.5%	0.000
	High direct bilirubin	7	12.5%	
Total protein status	Low total protein	10	17.9%	0.001
	Normal total protein	32	57.1%	
	High total protein	14	25.0%	
	Low albumin	33	58.9%	
Albumin status	Normal albumin	20	35.7%	0.000
	High albumin	3	5.4%	
	Low Na	42	75.0%	
Na status	Normal Na	13	23.2%	0.000
	High Na	1	1.8%	
	Low K	16	28.6%	
K status	Normal K	31	55.4%	0.001
	High K	9	16.1%	
	Low AST	0	0.0%	
AST status	Normal AST	31	55.4%	0.423
	High AST	25	44.6%	
	Low ALT	0	0.0%	
ALT status	Normal ALT	29	51.8%	0.789
	High ALT	27	48.2%	
	Low ALP	0	0.0%	
ALP status	Normal ALP	28	50.0%	1.000
	High ALP	28	50.0%	
	Low ESR	0	0.0%	
ESR status	Normal ESR	2	3.6%	0.000
	High ESR	54	96.4%	
CRP status	Normal CRP	6	10.7%	0.000
	High CRP	50	89.3%	

**Figure 1:**
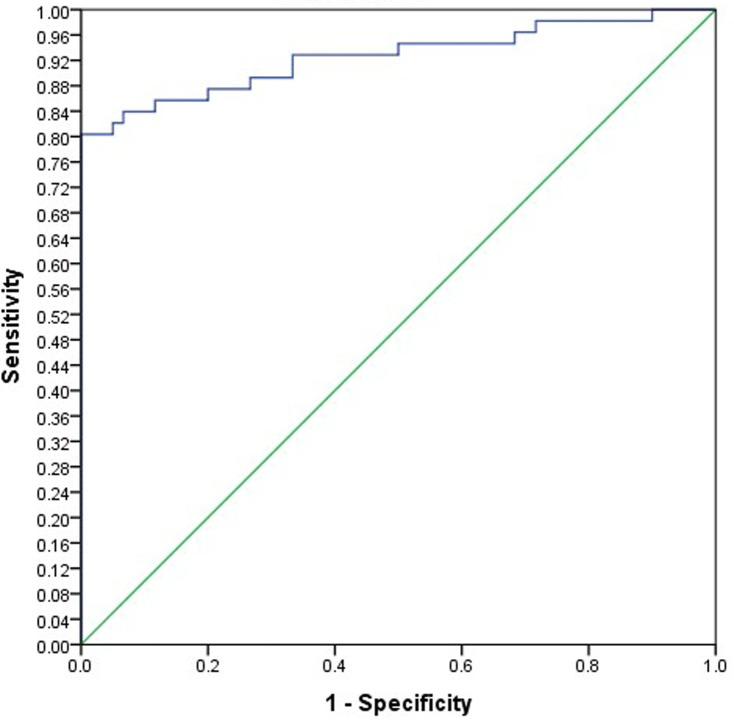
the receiver operating characteristic curve and area under the curve (ROC/AUC) of lymphocytes percentage

**Figure 2:**
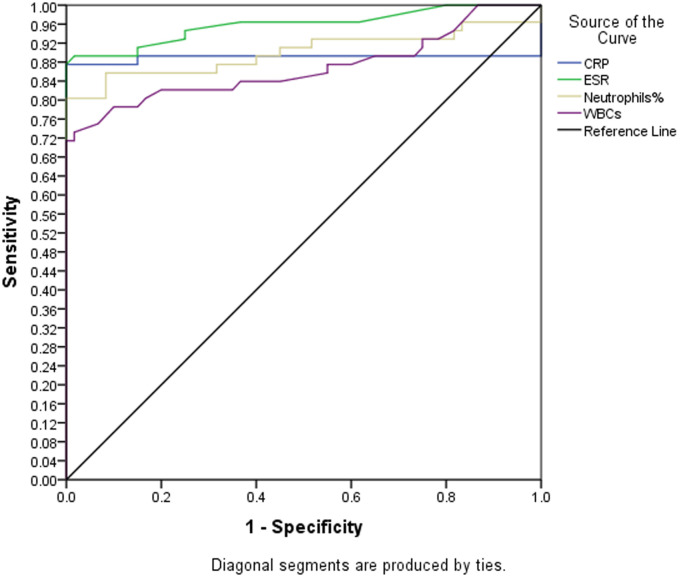
the receiver operating characteristic curve and area under the curve (ROC/AUC) of C-reactive protein (CRP), erythrocyte sedimentation rate (ESR), neutrophils and white blood cells (WBCs)

Analysis using Pearson correlation test showed an inverse relationship between lymphocytes and neutrophils (P=0.000) and between lymphocytes and WBCs (P=0.000). The receiver operating characteristic curve (ROC)/area under the curve (AUC) ensure the expellant result of lymphocytes percentage as a predictor with 92% AUC, neutrophils were 90% AUC and ESR 95.8% AUC. The good result recorded for CRP was 89% AUC and WBCs were 86.8% AUC. The percentage of detecting COVID-19 positive patient using RT-PCR is (98%), for suspected individuals using ROC and the best cutoff for lymphocytes percentage (≤21.8), neutrophils (≥67.7), ESR (≥37.5), CRP (≥6.2) and WBCs (≥7.15). The true positive and false positive presented in Table 3, Figure 1 and Figure 2.

**Table 3:** predictive values from receiver operating characteristic curve and area under the curve (ROC/AUC)

Effect variables	Urea under ROC/AUC	Best cutoff	TP	FP
Lymphocytes	92.6% excellent area	Less than 21.8	80%	0.0%
CRP	89% good area	More than 6.2	88%	1.5%
ESR	95.8% excellent area	More than 37.5	88%	0.0%
Neutrophils	90% excellent area	More than 67.7	80%	0.0%
WBCs	86.8% good area	More than 7.15	73%	0.0%

## Discussion

The rRT-PCR test remains the gold standard method for the etiological diagnosis of SARS-CoV-2 infection. In addition to the demands and limitations of this technique even for developed countries, there is also a need for certified laboratories with expensive equipment and highly trained personnel [6]. The challenges are even more for developing countries and those with limited health resources due to shortage of specialized laboratories and/or increased reagents cost. These limitations were translated in very limited numbers of qualified molecular laboratories capable of testing, leading to an extremely long waiting list of COVID-19 suspected patients. Furthermore, the main objective of the present study is to find sensitive and predictive blood markers so as to help in prioritizing those with high vulnerability and susceptibility of being infected.

Although, many researches focus on the correlation of blood markers and the severity of the disease and/or its outcome, not many studies investigate the role of blood markers in prioritizing rRT-PCR for COVID-19 suspected patients in countries with limited health resources. If such markers turn out to be of high sensitivity and specificity in predicting patients with COVID-19 infection, it will reduce the long waiting list of molecular testing, improving the morbidity and reducing the mortality with COVID-19 in developing countries. In our study, 4 markers (lymphocytes, neutrophils, CRP and ESR) were potentially important in predicting who will show positive PCR in COVID-19 (Table 3). Most of these markers were reported in other studies showing good correlation between the blood marker and the disease progression and outcome [7]. In our study, the results showed WBCs normal median range in most of COVID-19 patients on their admission day (Table 2). This result of WBCs was in complete accordance with what has been reported in the literature [8]. However, 78.6% of patients showed low lymphocyte counts (lymphopenia) and statically significant frequency distribution with a P-value=0.000. Comparing our result with the previous studies, our data showed even stronger correlation between the lymphopenia and COVID-19 infection with a predictor value of 92% (sensitivity and specificity) [8,9].

Lymphopenia was also observed in the previous two outbreaks that were caused by coronaviruses; sever acute respiratory syndrome (SARS) in 2003 and Middle East Respiratory Syndrome (MERS) in 2012. The pathogenesis of lymphopenia as recent studies revealed, include direct viral infection, immune mediated lymphocytes destruction and cytokine-mediated altered lymphocyte trafficking and sequestration [10,11]. The decline in lymphocytes count is used in evaluation of severity and outcome of COVID- 19 infection [12].

In addition to lymphocytes %, an increase in 3 other blood markers (neutrophils, CRP, ESR) showed high prediction for COVID-19 infection. Similar data was reported for CRP, ESR and neutrophils [8,13]. We observed no association between Hb, platelets and the disease (Table 2); this was in contrast to the study conducted by Chen *et al*. [14], which showed a significant association between low Hb, thrombocytopenia and COVID-19 patients. However, we acknowledge that our study suffers from a few limitations like the relatively limited number of patients and the absence of a controlled population, clinical signs which can help in discriminating between positive and negative COVID-19 patients.

## Conclusion

According to the present study, physicians in countries with limited testing resources may need to include blood markers (lymphocytes, neutrophils, CRP and ESR) to prioritize the patients with a high suspicion of having COVID-19 for rRT-PCR test based on these markers. If done it may reduce the false-negative number of COVID-19 patients and improve the clinical course of the disease by improving morbidity and reducing the mortality in those patients.

### What is known about this topic

- The gold standard diagnostic test for COVID-19 is identification of virus RNA using rRT-PCR technique;
- The molecular diagnosis required specialized molecular virology labs and highly trained personnel; both are limited in developing countries with low health resources.

### What this study adds

- The present study provides blood markers (lymphocytes %, neutrophils, CRP and ESR) that can be used to increase the predictively for the diagnosis of COVID-19 patients, which may help physicians to prioritize rRT-PCR suspected patients who really need to do confirmatory molecular test;
- The blood markers suggested by this study may help countries with limited health resources to maximize the benefit use of their limited diagnostic resources so as to reduce rRT-PCR false negative results; that may lead to decrease morbidity by early detection and isolation of confirmed COVID-19 positive cases.

## References

[1] Coronaviridae Study Group of the International Committee on Taxonomy of Viruses (2020). The species severe acute respiratory syndrome-related coronavirus: classifying 2019-nCoV and naming it SARS-CoV-2. Nat Microbiol.

[2] Islam MT, Talukder AK, Siddiqui MN, Islam T (2020). Tackling the pandemic COVID-19: the Bangladesh perspective Preprint.

[3] Lippi G, Plebani M (2020). Laboratory abnormalities in patients with COVID-2019 infection. Clin Chem Lab Med CCLM.

[4] Cohen J, Kupferschmidt K (2020). Countries test tactics in war against COVID-19. American Association for the Advancement of Science.

[5] Ferrari D, Motta A, Strollo M, Banfi G, Locatelli M (2020). Routine blood tests as a potential diagnostic tool for COVID-19. Clin Chem Lab Med CCLM.

[6] Tiede I, Fritz G, Strand S, Poppe D, Dvorsky R, Strand D (2003). CD28-dependent Rac1 activation is the molecular target of azathioprine in primary human CD4+ T lymphocytes. J Clin Invest.

[7] Velavan TP, Meyer CG (2020). Mild versus severe COVID-19: laboratory markers. Int J Infect Dis.

[8] Huang C, Wang Y, Li X, Ren L, Zhao J, Hu Y Clinical features of patients infected with 2019 novel coronavirus in Wuhan, China. The lancet. 2020;.

[9] Xu X-W, Wu X-X, Jiang X-G, Xu K-J, Ying L-J, Ma C-L (2020). Clinical findings in a group of patients infected with the 2019 novel coronavirus (SARS-Cov-2) outside of Wuhan, China: retrospective case series. bmj.

[10] Ko J-H, Park GE, Lee JY, Lee JY, Cho SY, Ha YE (2016). Predictive factors for pneumonia development and progression to respiratory failure in MERS-CoV infected patients. J Infect.

[11] Azkur AK, Akdis M, Azkur D, Sokolowska M, van de Veen W, Brüggen M-C (2020). Immune response to SARS-CoV-2 and mechanisms of immunopathological changes in COVID-19. Allergy.

[12] Ruan Q, Yang K, Wang W, Jiang L, Song J (2020). Clinical predictors of mortality due to COVID-19 based on an analysis of data of 150 patients from Wuhan, China. Intensive Care Med.

[13] Ling W (2020). C-reactive protein levels in the early stage of COVID-19. Med Mal Infect.

[14] Zhang Y, Xiao M, Zhang S, Xia P, Cao W, Jiang W (2020). Coagulopathy and antiphospholipid antibodies in patients with Covid-19. N Engl J Med.

